# Hyaluronan-Conjugated Carbon Quantum Dots for Bioimaging Use

**DOI:** 10.1021/acsami.0c20088

**Published:** 2020-12-23

**Authors:** Bedia Begüm Karakoçak, Amine Laradji, Tina Primeau, Mikhail Y. Berezin, Shunqiang Li, Nathan Ravi

**Affiliations:** † Department of Ophthalmology and Visual Sciences, Washington University in St. Louis, St. Louis, Missouri 63110, United States; ‡ Veterans Affairs Medical Center, St. Louis, Missouri 63106, United States; § Department of Medicine, Washington University School of Medicine, St. Louis, Missouri 63110, United States; ∥ Department of Radiology, Washington University School of Medicine, St. Louis, Missouri 63110, United States

**Keywords:** bioimaging, carbon quantum dots, hyaluronic acid, CD44 receptors, tumor imaging, cancer, breast cancer

## Abstract

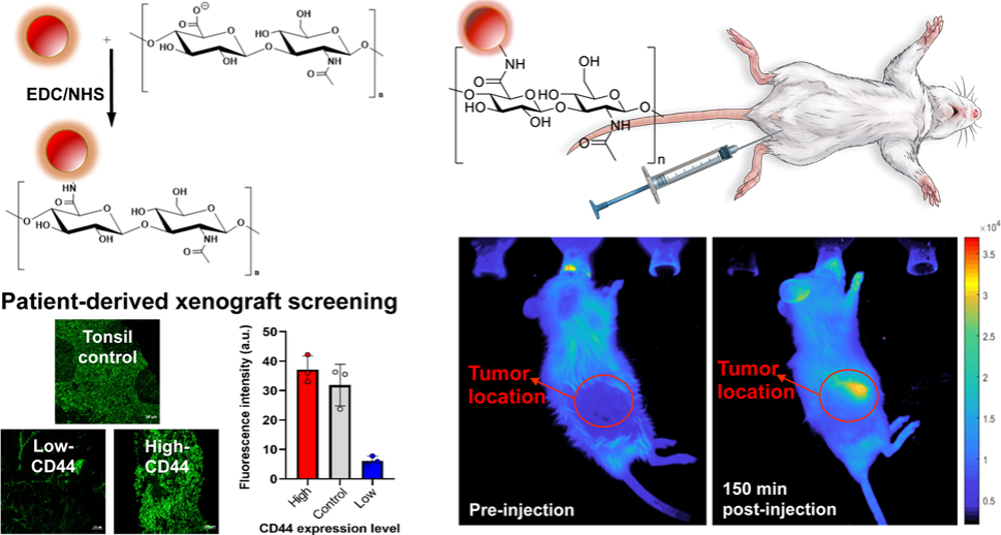

This work demonstrates the application of hyaluronan-conjugated nitrogen-doped carbon quantum dots (HA-nCQDs) for bioimaging of tumor cells and illustrates their potential use as carriers in targeted drug delivery. Quantum dots are challenging to deliver with specificity, which hinders their application. To facilitate targeted internalization by cancer cells, hyaluronic acid, a natural ligand of CD44 receptors, was covalently grafted on nCQDs. The HA-nCQD conjugate was synthesized by carbodiimide coupling of the amine moieties on nCQDs and the carboxylic acids on HA chains. Conjugated HA-nCQD retained sufficient fluorescence, although with 30% lower quantum efficiency than the original nCQDs. Confocal microscopy showed enhanced internalization of HA-nCQDs, facilitated by CD44 receptors. To demonstrate the specificity of HA-nCQDs toward human tumor cells, patient-derived breast cancer tissue with high-CD44 expression was implanted in adult mice. The tumors were allowed to grow up to 200–250 mm^3^ prior to the injection of HA-nCQDs. With either local or systemic injection, we achieved a high level of tumor specificity judged by a strong signal-to-noise ratio between the tumor and the surrounding tissue *in vivo*. Overall, the results show that HA-nCQDs can be used for imaging of CD44-specific tumors in preclinical models of human cancer and potentially used as carriers for targeted drug delivery into CD44-rich cells.

## Introduction

The noninvasive visualization of a cell/tissue/organ is a crucial tool for diagnosing and treating various diseases, including cancer. The last two decades have seen extensive research on multiple materials for cancer nanotheranostics,^1−6^ and carbon quantum dots (CQDs) specifically promising for tumor imaging.^4,7−9^ CQDs are promising bioimaging agents because of their intrinsic properties. Some of the desired properties of bioimaging agents that are unique to the CQDs can be listed as the wavelength-dependent excitation and emission,^10,11^ relatively long fluorescence lifetime,^12^ stability and solubility in water,^13,14^ and finally superior biocompatibility.^15^

CQD synthesis methods range from reasonably simple to complex.^4,10,12,16−18^ For clinical applications, the ideal synthesis would rely on commercially available reactants, a low number of synthetic steps with minimum purification effort, and high product yeild.^14,19,20^ The microwave-assisted synthesis method is considered one of the promising techniques, offering relatively shorter reaction times (in the order of minutes), lower energy consumption as it is a single-step synthesis method, and greater product efficiency.^14,21,22^ Recently, we have successfully used a domestic microwave oven to synthesize nitrogen-doped CQDs (nCQDs).^14^ The reaction used citric acid aliphatic diamines in water as raw materials and took less than 2 min to achieve high product yield. The synthesized nCQDs were efficient emitters above 600 nm, a highly desirable characteristic for distinguishing their emissions from the tissue autofluorescence.^4,23^ To yield high fluorescence above 600 nm, we optimized the pyrolysis reaction time, citric acid, and aliphatic diamines molar ratio, and the reactant (citric acid and aliphatic diamines) concentrations.^14^

For cancer imaging applications, an imaging agent should be able to target specific receptors at the tumor site. In this work, we focused on a cluster of differentiation 44 (CD44) receptors. Being expressed in a large number of mammalian cell types, CD44 receptors are reported to be overexpressed in many pathologic conditions.^24−26^ In the eye, for example, pathological conditions include proliferative vitreoretinopathy, where retinal pigment epithelial (RPE) cells rapidly proliferate in the subretinal space.^27−29^ These rapidly dividing RPE cells expressing more CD44 receptors than their stationary states.^30,31^ On the other hand, tumor cells with epithelial origin have been reported to exhibit significantly higher CD44 receptors than healthy cells.^26,32^

Hyaluronic acid (HA), a natural biopolymer that participates in wound healing^33^ while acting as a reactive oxygen species (ROS) scavenger,^34^ is known to be the natural ligand for CD44 receptors.^35−38^ Because uncoated nCQDs lack specificity for the target tumor,^20,23^ in this study, we coated nCQDs with HA to test their affinity toward CD44 receptors *in vitro* and *in vivo*. In the *in vitro* applications, we tested the biocompatibility of HA-coated nCQDs with two different cell lines: (noncancerous) ARPE-19 and (cancerous) chinese hamster ovary (CHO). We quantified the viability of the cells, the apoptotic signal generated by the dying cells, and the ROS production within the cells which were exposed to both uncoated and HA-coated nCQDs. We found that HA significantly enhanced the biocompatibility of nCQDs. In the *in vivo* experiments, we used CD44-rich tumors derived from human patients implanted in the NSG mice. The imaging constructs were injected either subcutaneously or intravenously. The resulting target specificity, indicated by a local tumor-specific increase in fluorescence intensity, confirmed the targeting ability of HA-nCQDs and demonstrated its potential as a promising bioimaging agent for CD44-expressing tumors.

## Experimental Section

### Materials

All chemicals were used as received unless specified otherwise. Sodium hyaluronate (HA) (*M*_W_ = 20 kDa, cat# HA-20K-1) was obtained from Lifecore Biomedical (Chaska, MN). *N*-hydroxysuccinimide (NHS, 98% purity, cat# 130672), *N*-(3-dimethylaminopropyl)-*N*′-ethyl-carbodiimide hydrochloride (EDC, 98% purity, cat# E1769), ethylenediamine (EDA, 99% purity, cat# E26266), Rhodamine 6G (99% purity, cat# 252433), Dulbecco’s modified eagle medium/nutrient mixture F-12 Ham (DMEM/F12, cat# 51445C), trypsin–ethylenediaminetetraacetic acid solution (trypsin–EDTA solution, 10×, cat# T4174), heat-inactivated fetal bovine serum (FBS, cat# F4135), Triton X-100 (cat# X100-100ML), phosphate buffer saline (PBS, 10×, cat# P5493), 3-[4, 5 dimethyl-thiazoly-2-yl] 2–5 diphenyl tetrazolium bromide (MTT, cat#M5655), paraformaldehyde (PFA, 95% purity, cat# 158–127) were acquired from Sigma-Aldrich (St. Louis, MO). The citric acid (cat# 15547774) was obtained from J.T. Baker Chemical Company (Phillipsburg, NJ). SlowFade Gold Antifade Mountant with 4′,6-diamidino-2-phenylindole (DAPI, cat# S36938) was obtained from Thermo Fisher Scientific (St. Peters, MO). The primary rabbit polyclonal to CD44 antibody (cat# ab157107) and the corresponding secondary antibody-goat anti-rabbit IgG H&L (Alexa Fluor 488) (cat# ab1500077) were both purchased from Abcam (Cambridge, MA). ARPE-19 (Retinal pigment epithelial) human cell line (ATCC CRL¬ 2302), CHO hLT-B: R-hG1 cell line (ATTC CRL¬ 11965), and NIH/3T3 (ATCC CRL¬ 1658) mouse cell line were purchased from American Type Culture Collection (ATCC) (Manassas, VA). The apoptosis assessment assay: ApoTox-Glo Triplex assay (cat# G6320) and reactive oxygen species (ROS) detection assay: ROS Glo H_2_O_2_ Assay (cat# G8820) were both obtained from Promega Corporation (Madison, WI). The Spectra/Por dialysis membrane tubings with a molecular weight cutoff (MWCO) of 100–500 and 12,000–14,000 Da (Cat# 131084 and 11495849, respectively) were purchased from Cole-Parmer GmbH (Vernon Hills, IL).

### Synthesis of nCQDs with Desired Properties

The nCQD synthesis method is described explicitly in our previous work.^14^ Briefly, a stock solution of citric acid (12 w/v %) was prepared in deionized (DI) water. This solution (7.34 mL) was transferred to a 100 mL beaker; the final volume of 10 mL was ensured with an additional 2.66 mL of DI water. Next, 840 μL of EDA solution was introduced to the stock solution and then finally heated to be pyrolyzed in a microwave oven (General Electric, 1100 W) for 88 s. The microwave-pyrolysis product—the resulting brown solid crystals—was dissolved in DI water (3 mL). The solution was then dialyzed with a dialysis membrane (MWCO 100–500 Da) against DI water. This solution was finally lyophilized to recover the synthesized nCQDs.

### Conjugation of nCQDs with HA

An aqueous HA (1 w/v %) solution was prepared by dissolving the powder form of HA (0.10 g, 0.24 meq of COOH) in 10 mL DI water. The pH of the HA solution was adjusted to 4.5. EDC (0.45 g, 2.4 mmol) and NHS (0.14 g, 1.2 mmol) were added to the HA solution at 22 °C. After stirring for 1 h, the nCQD solution (0.10 g, 5 mL) was added to the HA solution, and the reaction mixture was continuously stirred overnight (for 18 h).

The primary amine groups of the nCQDs are to be cross-linked to the carboxyl groups of hyaluronic acid (HA) following carbodiimide chemistry. The reaction proceeds through a zero-length carboxyl to-amine cross-linking agent, EDC. EDC activates HA’s carboxyl groups for direct reaction with the primary amines of nCQD *via* amide bond formation. Under acidic conditions, the carboxylic acid attacks EDC, forming an O-acylisourea intermediate, which is highly reactive and short-lived in an aqueous environment. Because the active ester can be rapidly hydrolyzed, we added NHS to improve the activated ester’s stability and, thus, increase the yield of the product and decrease side reactions. The pH was adjusted to 8–9 to terminate the reaction. The excess (unreacted) EDC, NHS, and unconjugated nCQDs were removed from the reaction mixture through dialysis (MWCO 12,000–14,000) against DI water (three times), and the HA-conjugated nCQDs were finally recovered by lyophilization as a brownish solid.

To confirm the successful conjugation, nuclear magnetic resonance (^1^H NMR) spectra were recorded and compared for native HA, as well as unconjugated and HA-conjugated nCQDs. The ^1^H NMR spectra were obtained using a Varian Unity Inova 500 MHz system (Palo Alto, CA), with D_2_O as the solvent (25 °C).

### Characterization of nCQDs and HA-CQDs

We recorded the ultraviolet–visible (UV–Vis) absorption spectra of the nCQDs and the HA-nCQDs at 540 nm in DI water using Thermo Fisher Biomate 3 UV–Vis spectrophotometer. To calculate the molar absorptivities (ε) of the nCQDs and their HA-conjugated counterparts, both CQDs were first lyophilized. After lyophilization, CQDs in the solid form were redissolved in DI water at a concentration range of 0.0–1.0 mg/mL. Molar absorptivities were measured at 540 nm.

The fluorescence spectra of CQDs (in DI water) were obtained under ambient conditions with a SpectraMax Gemini EM microplate reader (excitation at 540 nm).

Three-dimensional (3D) excitation/emission maps of the CQDs were recorded to detect the carbon quantum dots’ homogeneity and possible fluorescent impurities, as detailed in our previous reports.^14,39^ Briefly, the measurements were carried out using a visible-near-infrared (Vis–NIR; 300–1600 nm) spectrofluorometer. The spectrofluorometer consists of an imaging spectrograph iHR320 and a diode array deep-cooled charge-coupled device detector synapse (the corresponding parts were obtained from Horiba Jobin Yvon Inc.). The samples were diluted with DI water until transparent, following sonication for 5 min before taking the measurements.

The quantum yield (QY) of the unconjugated and HA-conjugated nCQD samples were calculated following a standard technique published by Dr. Brouwer as a part of the International Union of Pure and Applied Chemistry technical report on measuring the photoluminescence QY in solution.^40^ The QYs of both unconjugated and HA-conjugated nCQDs in DI water (excitation at 540 nm) were determined. Rhodamine 6G was used as a reference in 10 mm quartz cuvettes, as described in our previous study.^14^ Finally, the integrated photoluminescence intensity (590–720 nm) *versus* the absorbance was plotted for both materials and compared to the reference dye (Rhodamine 6G) at different concentrations.

### Hydrodynamic Size and Zeta Potential Measurements with Dynamic Light Scattering Analysis

Hydrodynamic size and zeta potential measurements of the carbon quantum dots were carried out using a Zetasizer Nano ZS (Malvern, Westborough MA). Carbon quantum dots were dispersed in DI water, and the obtained solution was sonicated for 15 min to ensure a homogeneous dispersion. Carbon quantum dots solution was, next, filtered through a prerinsed sterile 0.22 μm filter and then equilibrated (for 5 min) at 25 °C. Three or more size measurements per carbon dot solution (both unconjugated and conjugated) were recorded.

### *In Vitro* Biocompatibility Assessments of the Carbon Quantum Dots with ARPE-19 Cells and CHO Cells

For biocompatibility measurements, the ARPE-19 and CHO cell lines were seeded at 2 × 10^4^ cells per well in a 96-well plate and grown to confluency—an optimized cell density in our previous studies specifically for biocompatibility tests.^41−43^ Next, the viability of cells in response to both unconjugated and conjugated nCQDs (the exposure concentration ranging from 0.01 to 0.6 mg/mL) was quantified using MTT assay as explicitly described in our previous studies.^41,42,44^ The relative amount of cells that initiated programmed cell death (apoptosis) and intracellular ROS amount were quantified (both relative to the negative controls, unexposed cells) using ApoTox-Glo and ROS Glo H_2_O_2_ assays, respectively, according to the manufacturer’s protocol. To measure the output obtained from both assay analyses, we used a luminescence-based microplate reader (Spectra Max 190 Microplate Reader, Molecular Devices). The measurements were carried out under ambient light to quantify the relative luminescence signal generated inside the cells.

In all biological and imaging tests, cells without exposure to nCQDs served as a negative control. All biological test results (viability, apoptotic cell signal, and intracellular ROS generation) were reported as relative results (normalized by the corresponding negative control group’s output), using ordinary two-way analysis of variance (ANOVA). The P* value was set to be less than 0.05 for a statistically acceptable significance level.

### *In Vitro* Imaging of nCQDs and HA-nCQDs

We investigated the carbon quantum dots’ intracellular distribution at a wide range of exposure doses (0.01–0.6 mg/mL). A confocal laser scanning microscope (CLSM) (Leica TCS-SP8) was used. The selected excitation wavelengths were 405, 532, and 635 nm. The nCQD detection range was 435–700 nm. The image processing was conducted using Leica Application Suite X (Leica, Buffalo Grove, IL) and IMARIS image analysis software (Bitplane AG, Zurich, Switzerland).

ARPE-19, CHO, and NIH 3T3 cells were seeded in CELLview plates at an optimized cell density of 5 × 10^4^ cells per well.^14,39,42^ The cells were allowed to grow for 24 h prior to exposure to the carbon quantum dots. Then, the nCQDs and HA-nCQDs were added to separate wells. After a 24 h exposure period, the cells were fixed with 4% PFA and washed with 1× PBS. The fixed cells were then incubated in 0.1% Triton X-100 with PBS for 10 min to permeabilize the cell membrane. The plates were then incubated with blocking buffer for 30 min at room temperature prior to the nuclei staining step. We used SlowFade Gold Antifade Mountant with DAPI (diluted 1:100 in 1× PBS) to stain the nuclei. The cells were kept at 4 °C overnight and then transferred to the confocal microscope for visual analyses. We used 405 nm (for DAPI—nuclei staining) and 405, 532, and 635 nm (for detecting the carbon quantum dots) as the laser wavelengths.

### Tissue Microarray Handling and Patient-Derived Xenograft Preparation for CD44 Staining

Tissue microarrays were constructed from 35 different formalin-fixed paraffin-embedded (FFPE) tumor samples from basal subtype breast cancer patient-derived xenografts (PDX), as explained previously.^45,46^ Cores were arrayed into a new recipient paraffin block with control tissues. The Supporting Information provides more information about establishing and characterizing patient-derived tumor xenografts.

### Immunofluorescent Staining of the PDX Slides

The slides were deparaffinized with dH_2_O. The slides were pretreated in TRIS EDTA with Tween 20 at pH 9.0 in a pressure cooker for 3 min. Next, the slides were treated with 20% inactivated donkey serum. The primary rabbit anti-CD44 antibody was added to the slides in a 1:200 dilution ratio, and the slides were incubated overnight at 4 °C. Next, the slides were washed with 1× PBS four times, for 15 min per wash. The goat anti-rabbit IgG H&L (Alexa Fluor 488) was added in 1:250 dilution, and the slides were incubated at room temperature for 2 h. The slides were then washed with 1X PBS at least three times, 15 min per each wash. Finally, the coverslips for confocal imaging were placed on slides with SlowFade Gold Antifade with DAPI staining.

### *In Vivo* Imaging of nCQDs and HA-nCQDs

All animal procedures were reviewed and approved by the Institutional Animal Care and Use Committee at Washington University in St. Louis. After CD44 receptor expression screening, a relatively high-CD44 receptor-expressing breast cancer PDX was selected (WHIM4). Six- to eight-week old NSG female mice were obtained from Jackson Laboratory (Bar Harbor, ME). The WHIM4 cells were injected into the NSG mice, and the tumors were grown up to 200–250 mm^3^ in volume.

The injection site was shaved before nCQD injection. nCQDs and HA-nCQDs were injected into the tumor-bearing mice, either intravenously or subcutaneously, on the upper back at a dose of 0.4 mg/kg. Animals were imaged prior to subcutaneous and intravenous injections and then again at 15 and 30 min post-subcutaneous injection and 150 min post-intravenous injection, using the pearl small-animal NIR fluorescence (λ_ex/em_: 510–630 nm/600–700 nm) imaging system (LICOR Biosciences, Nebraska).

## Results and Discussion

### Conjugation of HA with nCQDs

**Figure 1:**
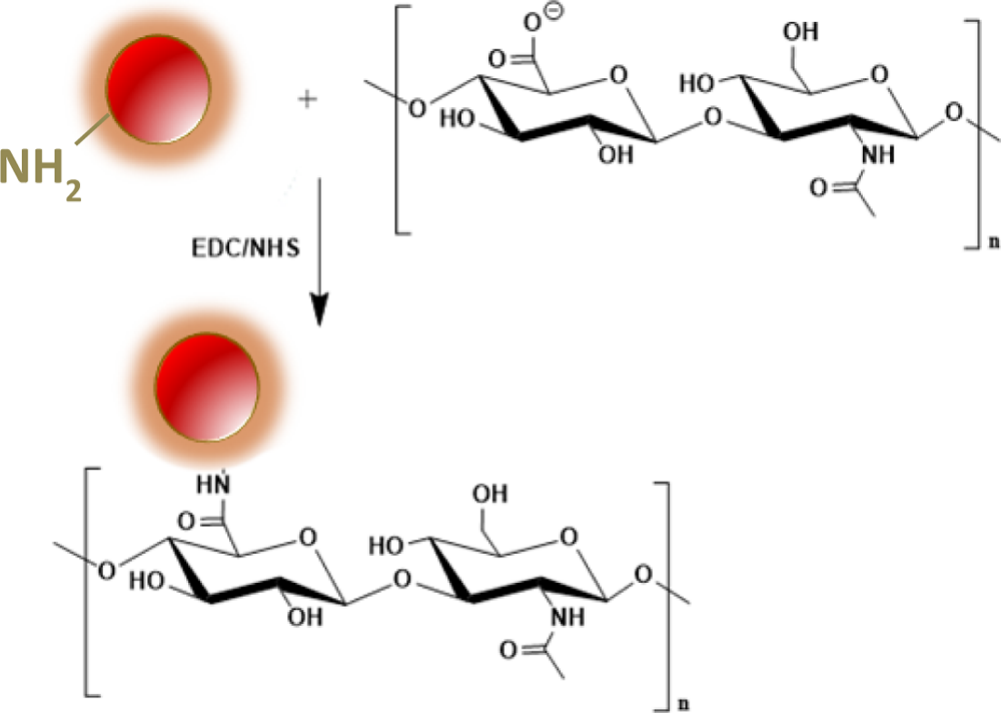
Reaction scheme of conjugating nCQD with hyaluronic acid.

The nCQDs were conjugated with HA to enable them to target cells with CD44 receptors. In addition, HA conjugation was expected to increase the biocompatibility of the nCQDs.^47^ An EDC coupling method^48^ was used to form amide bonds between the carboxylic groups of HA and the amine groups of nCQDs (Figure 1). Because nCQDs contain both amino and carboxyl groups, amide bonds may also be formed between two nCQDs. To minimize cross-linking between nCQDs, the carboxylic activators EDC and NHS were first reacted with HA for 1 h prior to adding nCQDs.

**Figure 2:**
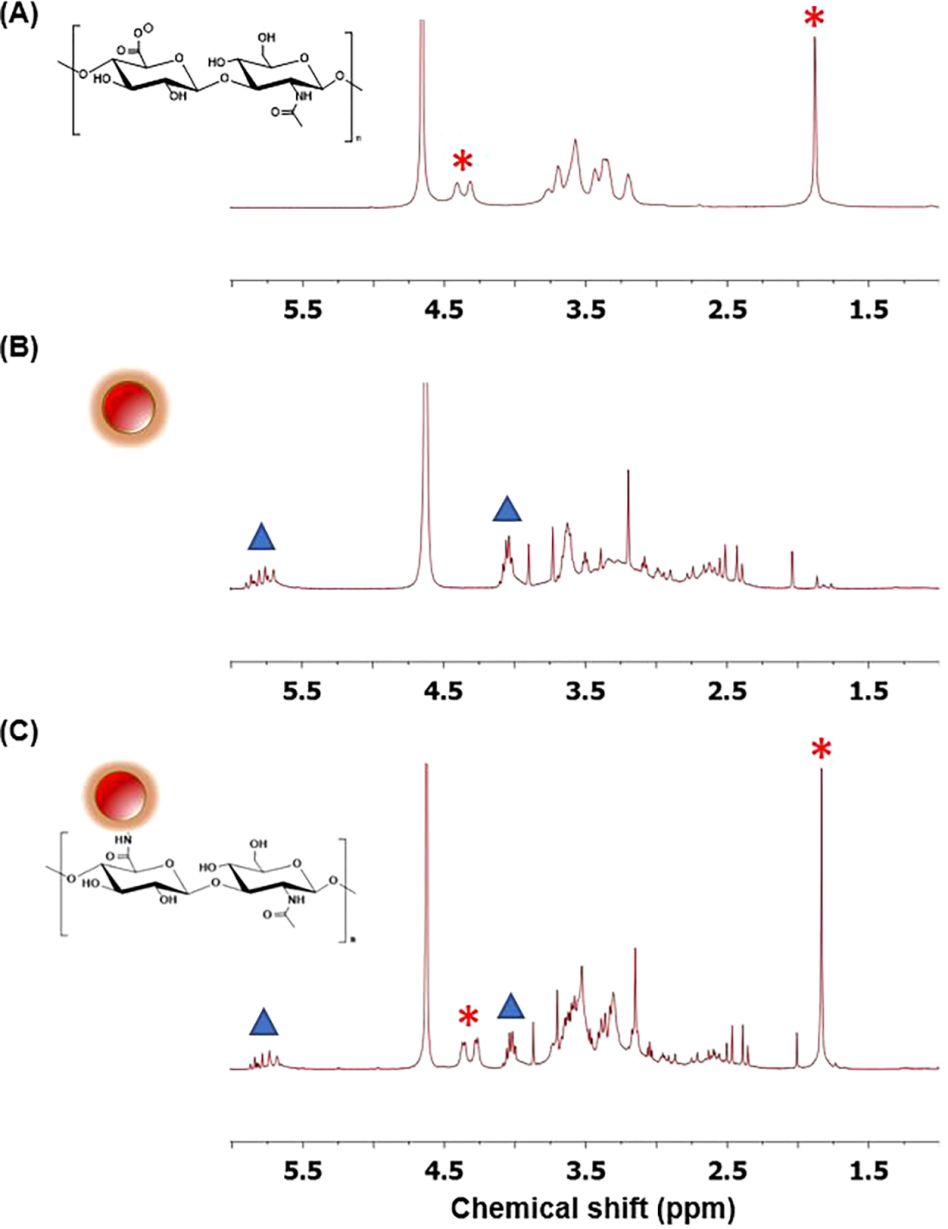
^1^H NMR spectra of (A) HA, (B) nCQDs, and (C) HA-nCQD conjugate confirming the presence of HA and nCQD peaks in the HA-nCQD conjugate.

To confirm the conjugation, ^1^H NMR was used to detect the presence of HA on nCQDs (Figure 2). The peaks at 1.88 and 4.3–4.4 ppm (labeled with asterisks in Figure 2A) indicate the presence of HA, and the peaks at 4.05 and 5.7–5.9 ppm (marked with triangles in Figure 2B) belong to nCQDs. In the ^1^H NMR spectrum of the HA-nCQD conjugate (Figure 2C), we detected peaks corresponding to both HA and nCQD. As a result, the ^1^H NMR spectra support the successful conjugation of HA to nCQDs.

### Characterization of Unconjugated and Conjugated nCQDs

**Figure 3:**
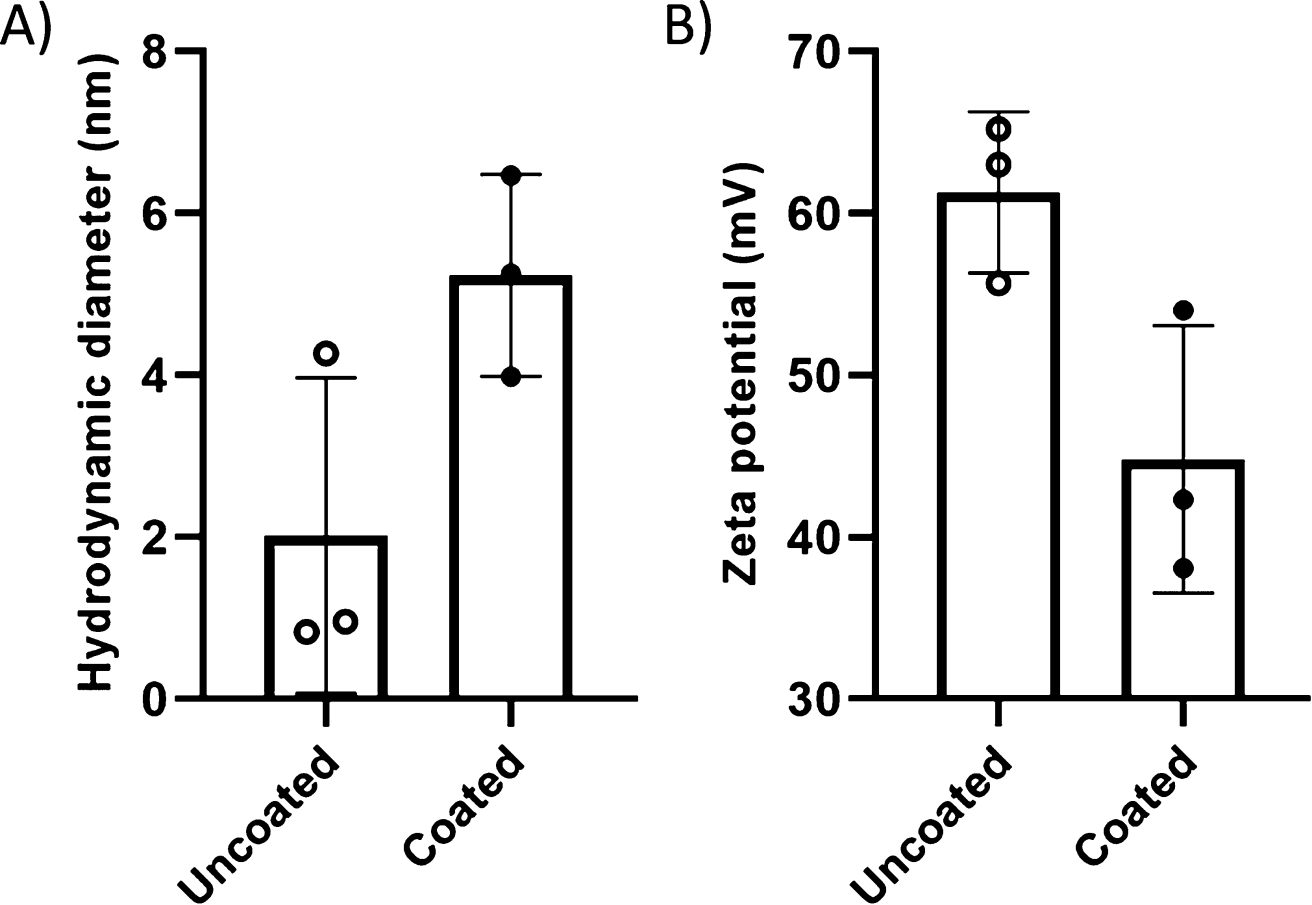
DLS analysis of nCQDs consisting of (A) hydrodynamic particle diameter and (B) zeta potential measurements. With HA coating, although the zeta potential of the nCQDs has decreased, 44.8 mV is still considered a high enough potential for particle stability. No aggregates or precipitates were observed in the solution after HA conjugation to nCQDs.

Dynamic light scattering (DLS) analysis showed the hydrodynamic sizes of the unconjugated nCQDs and HA-nCQDs in DI water were 2.01 ± 1.95 and 5.24 ± 1.25 nm (Figures 3A and S1), respectively. Zeta potential analysis showed that with HA coating, the zeta potential of nCQDs decreased from 61.3 ± 7.74 to 44.8 ± 8.04 mV (Figures 3B and S2).

In the next step, we calculated molar absorptivities (ε) at 540 nm of the nCQDs and their HA-conjugated counterparts using absorption spectra. The slopes of the absorption *versus* concentration revealed that the molar absorptivities of nCQDs and HA-nCQDs were 2.35 × 10^6^ and 1.65 × 10^6^ M^–1^ cm^–1^, where the molar absorptivity of the HA-conjugated nCQDs was about 30% less than their unconjugated counterparts (Figure S3).

**Figure 4:**
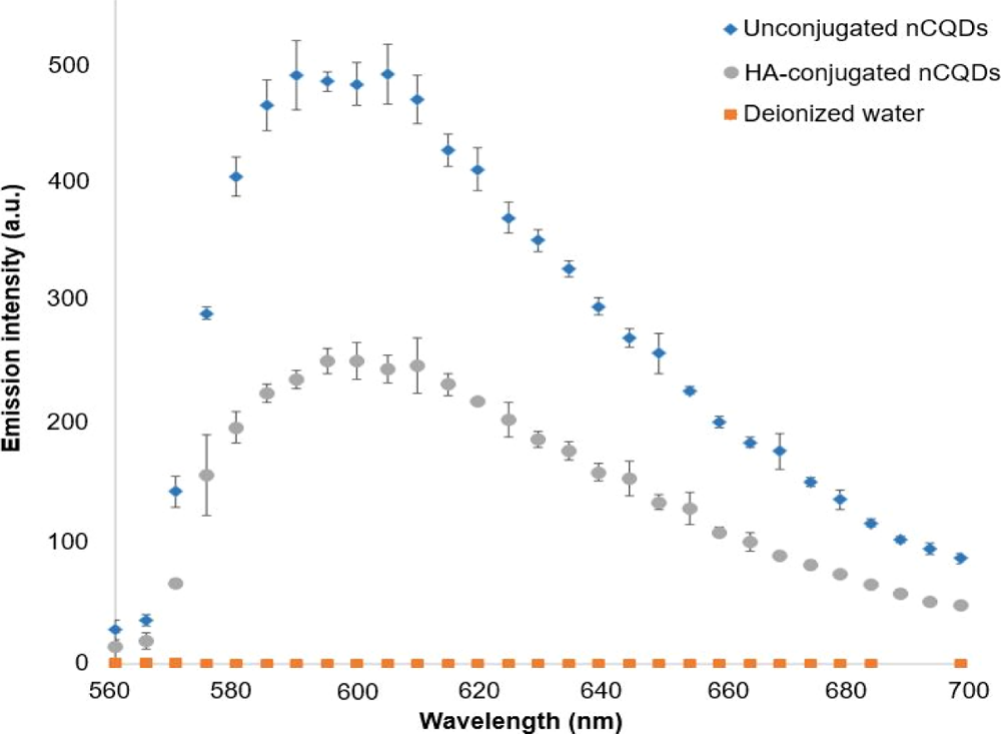
PL emission spectra of unconjugated nCQDs with citric acid to aliphatic diamines ratio of 2.0, and their HA-nCQD conjugates. λ_excitation_ = 540 nm.

As a part of the characterization analysis, we compared the fluorescence spectra of nCQDs and HA-nCQD conjugates recorded at 540 nm excitation in DI water (Figure 4). The emission peak of the HA-nCQD conjugates remained at around the same wavelength as the nCQDs before conjugation, which suggests that the chemical conjugation did not alter the fluorescence behavior of the nCQDs at this specific wavelength. The PL intensity of the HA-nCQD conjugates was about half of that of the native nCQDs at the same concentration, consistent with a 1:1 M conjugation ratio of HA and nCQDs.

**Figure 5:**
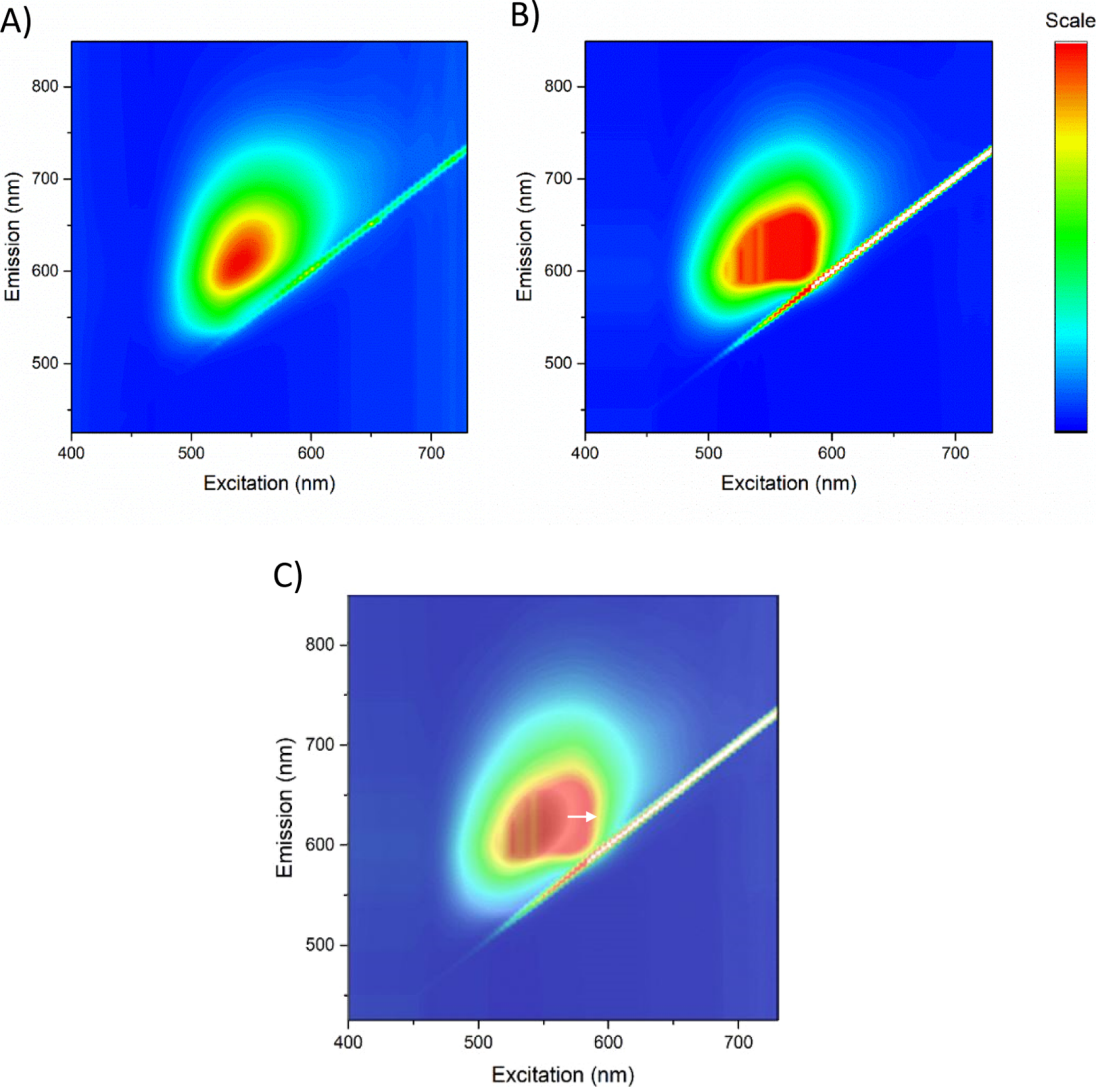
3D fluorescence excitation-emission spectroscopy of (A) nCQDs, from our previous study,^14^ and (B) HA-nCQDs. (C) Overlay of (A,B), indicating a redshift (marked with white arrow) in the emission characteristics of the HA-nCQDs. Although the excitability and the range for emission characteristics did not significantly differ between the two CQDs, the broader nonhomogenous shape of the emission peak in HA-nCQDs indicates heterogeneity among the emitters.

Next, we recorded fluorescence excitation-emission spectra to investigate the excitation wavelength’s effect on the emission of nCQDs and HA-nCQDs and resolved a multicomponent fluorescent mixture. The resulting 3D maps showed that both nCQDs and HA-nCQDs exhibit a typical^14,39^ excitation-dependent fluorescence characteristic (Figure 5). In the case of nCQDs (Figure 5A), a single symmetrical emission shape indicates a single emitter. In contrast, HA-nCQDs (Figure 5B) showed a nonsymmetrical and heterogeneous emission peak indicating the presence of several emitters. This finding reflects the varying conjugation degree among the different-sized nCQDs and the HA polymer.

Finally, we calculated the QY % of both unconjugated and HA-conjugated nCQDs in DI water. As we previously reported, the QY % of our nCQDs was 15.0% when measured at an excitation of 540 nm.^14^ Here, we found QY to be 10.5% for the HA-nCQDs. This loss in the QY (approximately 30%) could be explained by the redshift in the emission peak (Figure 5C) as a result of the increase in the size of the fluorophore (Figure 3A). Another explanation is possible partial quenching from the organic ligand; in this case, it is HA, which leads to a change in the surface charge of the nCQDs, as demonstrated in zeta potential measurements (Figure 3B). Our results agree with previous findings, where it was reported that PL peak redshifts due to the changes in the surface charge.^10^ This change in surface charge, which is induced by protonation–deprotonation, causes partial quenching, thus a decrease in the QY.^49,50^

### *In Vitro* Biocompatibility Testing of nCQDs and HA-nCQDs

In ocular clinical applications, intravenously introduced nanoparticles must pass the blood-retina barrier to access ocular tissues.^51^ In a typical ocular clinical application that involves targeted drug delivery to the dysfunctional RPE, the retinal epithelial cells play a central role.^52,53^ The dysfunctional RPE can lead to vascular leakage, which ultimately can threaten vision.^54^ With this information in mind, we investigated nCQDs’ toxicity to retinal (ARPE-19) cells. With cancer theranostics in mind, we evaluated the nCQDs’ compatibility with CHO cells.

To assess toxicity, we used three different independent end-point assays: MTT assay for viability assessment, ApoTox-Glo Triplex assay for apoptotic cell percentage quantification, and direct measurement of ROS with ROS Glo H_2_O_2_ assay as explained in the Methods section. The LD_50_ of nCQDs was previously found to be above 0.6 mg/mL,^14^ which was the highest tested exposure concentration for this study (Figures S4 and 5A). Up to this concentration, less than 50% of the ARPE-19 cells initiated apoptotic cell death (Figures S4 and 5B) but induced ROS generation (Figures S4 and 5C). Interestingly, it was found that the intracellular ROS level in CHO cells was significantly higher than the ARPE-19 cells. Despite the presence of considerably higher intracellular ROS levels, CHO cells’ viability was not hindered even at the highest exposure dose of the nCQDs (Figure S5). This tendency can be explained by cancer cells’ endurance to higher ROS levels than the healthy cells.^14,42,55^ Heightened metabolism rates and structural dysfunction in mitochondrion have been associated with elevated ROS levels in tumor cells.^56^

Finally, it is essential to note that the HA-nCQDs showed enhanced biocompatibility results for both cell lines tested. Both the apoptotic cell signal generated by the cells and the intracellular ROS concentration was decreased significantly for both ARPE-19 and CHO cells.

### *In Vitro* Imaging of nCQDs and HA-nCQDs

**Figure 6:**
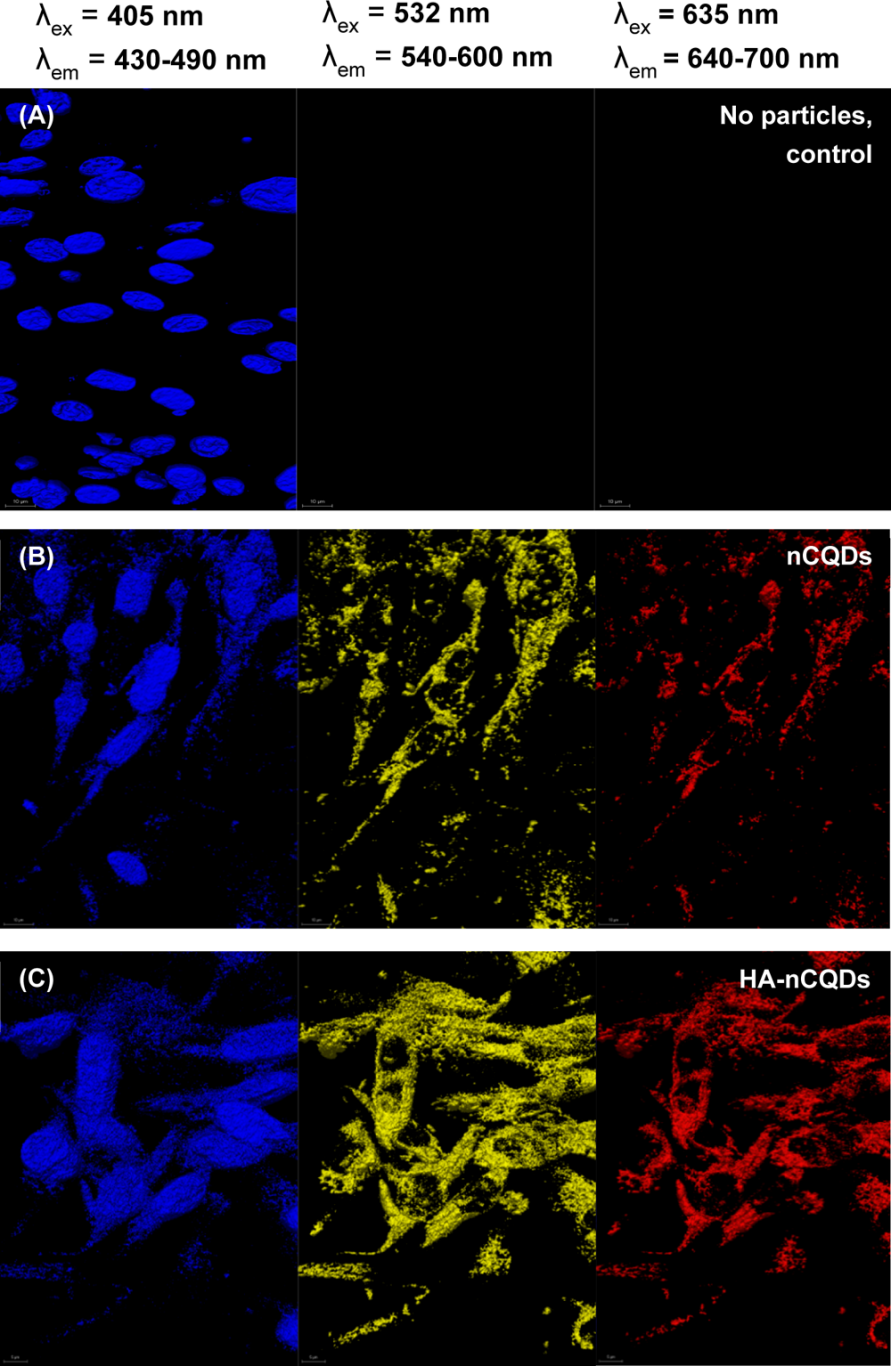
Confocal microscopy images of ARPE-19 cells exposed to 0.6 mg/mL nCQDs, with and without HA conjugation. The cell nuclei were stained with DAPI. The scale bars are 10 μm.

**Figure 7:**
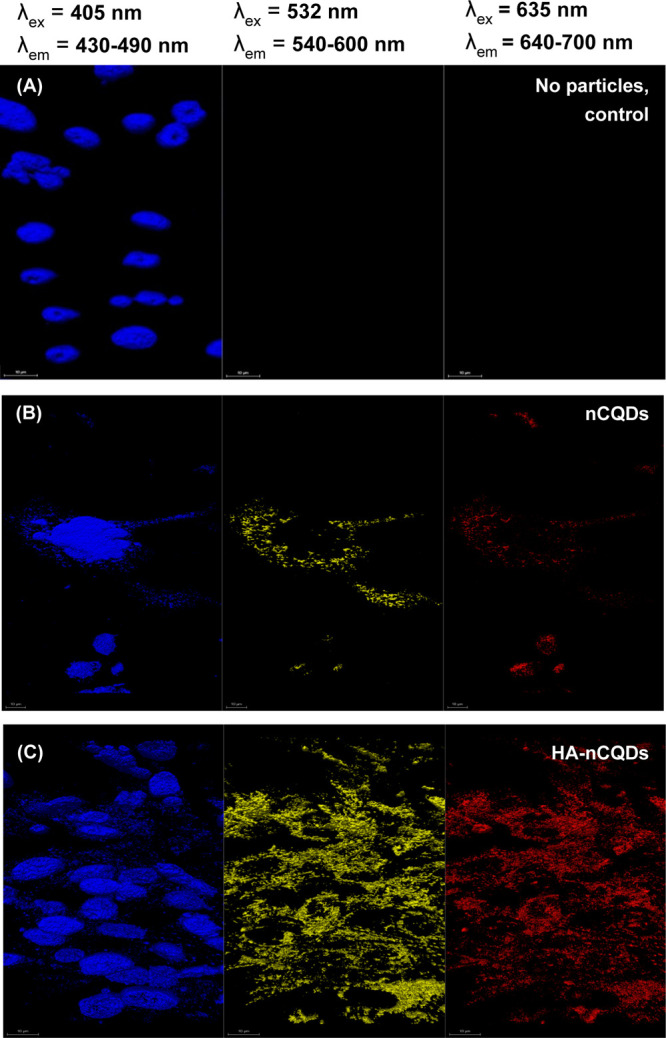
Confocal microscopy images of CHO cells exposed to 0.6 mg/mL nCQDs, with and without HA conjugation. The cell nuclei were stained with DAPI. The scale bars are 10 μm.

We investigated the intracellular presence of both unconjugated and conjugated nCQDs using a CLSM (LEICA TCS-SP8) (see Methods). ARPE-19 and CHO cells were used for the *in vitro* imaging analysis. The carbon quantum dots’ intracellular presence was successfully detected by fluorescence imaging because of their excitation-dependent emission characteristics (Figure 5). We have previously shown that nCQDs also fluoresce when excited at 635 nm,^14^ unlike previous reports where the same synthesis method was used, but the nCQDs were excitable only up to an excitation wavelength of 488 nm.^22,57^ Our optimization of the synthesis method revealed that at a high amine-to-acid ratio, in this case, NH_2_/COOH molar ratio of 2.0 plays a crucial role in red emissivity. We previously hypothesized that C=N bonds and pyrrolic N are the keys to obtain red-emissive nCQDs.^14^ The HA-nCQDs were also excitable up to 640 nm, and no signal loss was observed (Figures 4 and 5). In both high CD44-expressing cell lines tested, the HA conjugation significantly increased the intracellular concentration of nCQDs (Figures 6C and 7C). To test if the internalization was through CD44 receptor-mediated endocytosis, we pretreated both cell types with excess hyaluronic acid before nCQD exposure. With the pretreatment of excess HA, the CD-44 receptors were blocked. It was observed that when the CD44 receptors are blocked, the amount of internalized HA-nCQDs significantly decreased (Figure S6). Therefore, it can be inferred that HA not only enabled CD44 receptor-targeted delivery but also increased the number of nCQDs that entered the cells.

To further confirm the selectivity of HA-nCQDs and to demonstrate that the internalization is, in fact, through CD44 receptor-mediated endocytosis, unconjugated and HA-conjugated nCQDs were exposed to NIH 3T3 cells, which exhibit significantly less CD44 receptors than ARPE-19 cells and CHO cells with high CD44-expression on their cell membrane.^26,30,31,42,58^

The intracellular concentration of HA-nCQDs in NIH 3T3 cells (Figure S7C) was significantly less than the ARPE-19 (Figure 6C) and CHO cells (Figure 7C). This finding confirms facilitated entrance of the HA-nCQDs is indeed through the CD44 cell-receptor-mediated internalization for HA.

### Tissue Microarray Screening

**Figure 8:**
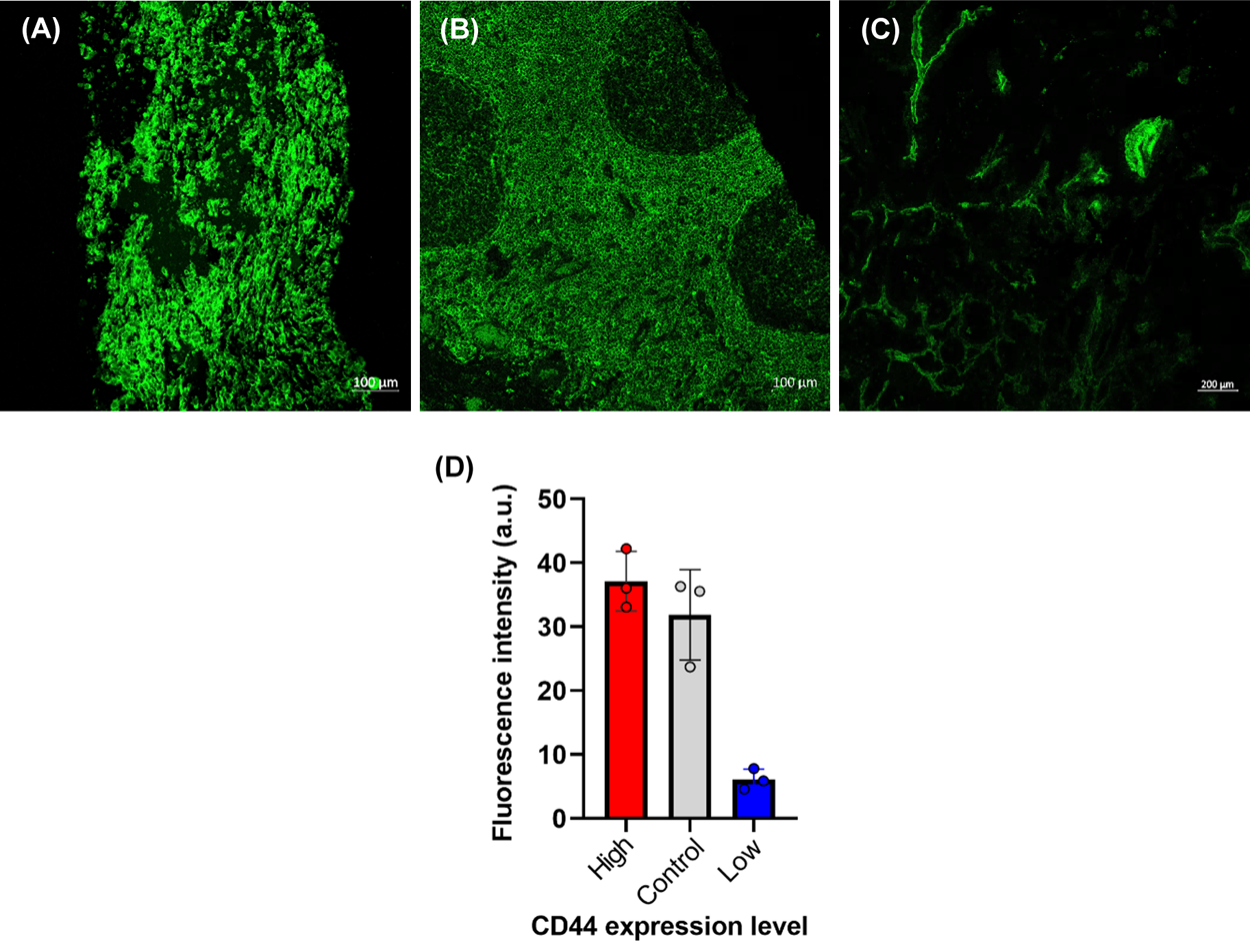
Tissue screening for CD44 expression. (A) High-CD44 expressing TMA sample. (B) Tonsil (Control for CD44 expression). (C) Low-CD44-expressing TMA sample. (D) Fluorescence intensity comparison of the CD44 expression of all three tissues. Scale bars in confocal images are 100 μm for (A) and (B) and 200 μm for (C).

The WHIM models exhibit remarkable genetic and phenotypic similarities with the human tumor from which they were derived.^45,46^ For the details of the establishment and characterization of the patient-derived xenograft, please refer to the Supporting Information. Briefly, at Washington University School of Medicine, patient-derived xenograft (PDX) models using breast cancer tissue and comparing the similarities between the original tumors and their xenografts were established. Thirty-five different models were screened for the CD44 expression with confocal microscopy imaging, and the results are presented in the Supporting Information (Table S1 and Figure S8). Examples of high-CD44 expressing (in terms of the fluorescence signal, 17% more than tonsil control) the tonsil section as a control for CD44 expressing tissue and low-CD44-expressing TMA samples (in terms of the fluorescence signal, 81% less than tonsil control) are shown in Figure 8.

### *In Vivo* Imaging of nCQDs and HA-nCQDs

**Figure 9:**
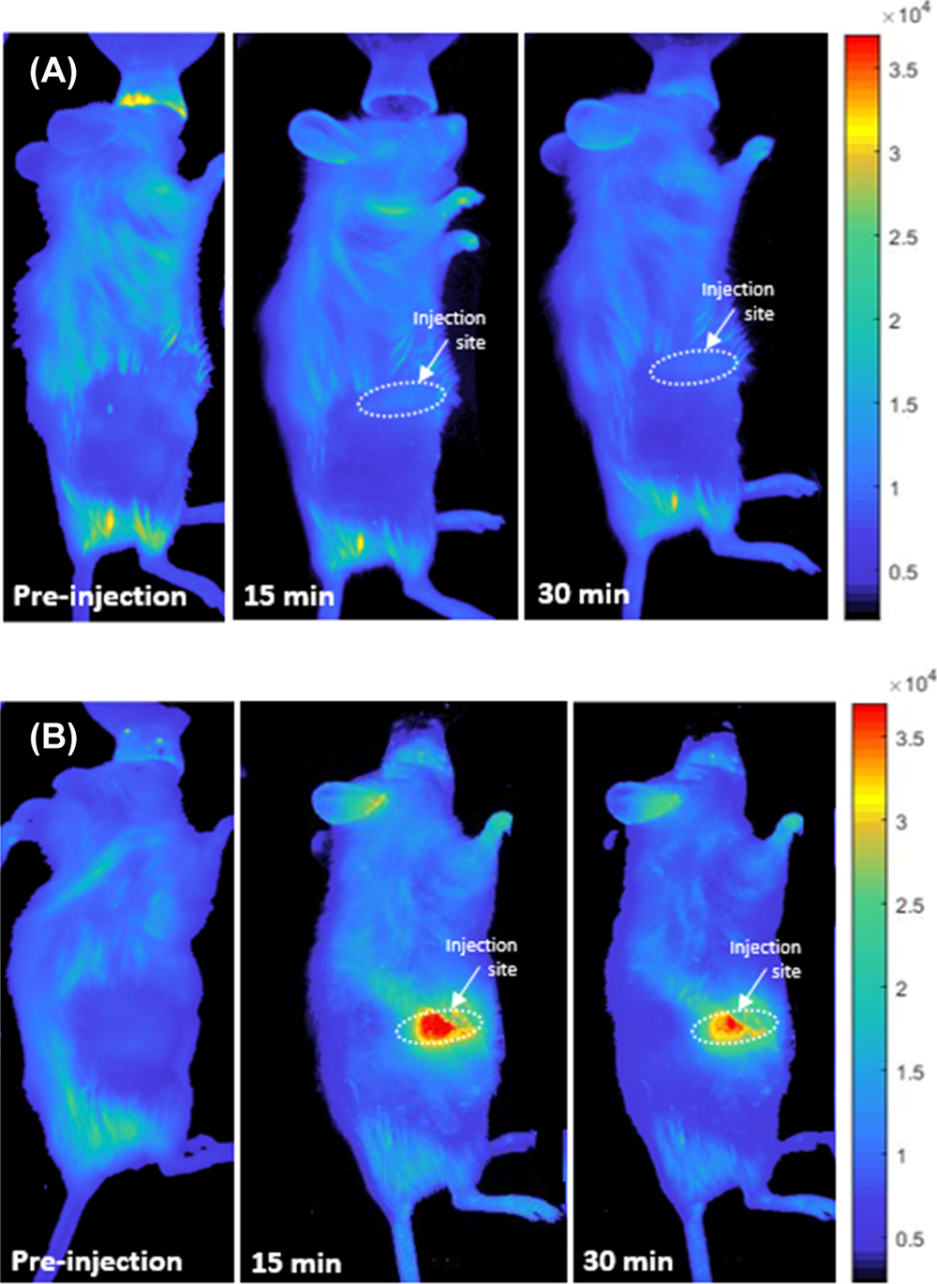
*In vivo* fluorescent images of mice injected with WHIM4 tumor cells after the tumor has grown up to 226.8 mm^3^ in volume. The images were obtained before and after subcutaneous injection of (A) nCQDs and (B) HA-nCQDs.

The feasibility of using carbon quantum dots for tumor imaging was evaluated *in vivo via* both subcutaneous and intravenous injections. HA-nCQDs were subcutaneously injected into one mouse, while another mouse received an nCQDs injection as a control sample. As shown in Figure 9, HA-nCQDs accumulated in the tumor site 15 min after injection, and the fluorescence signal from the HA-nCQDs was detectable at the tumor site (Figure 9B). In contrast, no fluorescence signal was detected in the mouse injected with nCQDs (Figure 9A), suggesting that hyaluronic acid coating of nanoparticles is critical for its ability to target cells overexpressing CD44 receptors. Because the tumor area was not targeted with nCQDs, the fluorescence signal from diffused nCQDs was not detectable. On the other hand, the HA coating allowed the nCQDs to concentrate in the tumor site, resulting in a high fluorescence signal.

**Figure 10:**
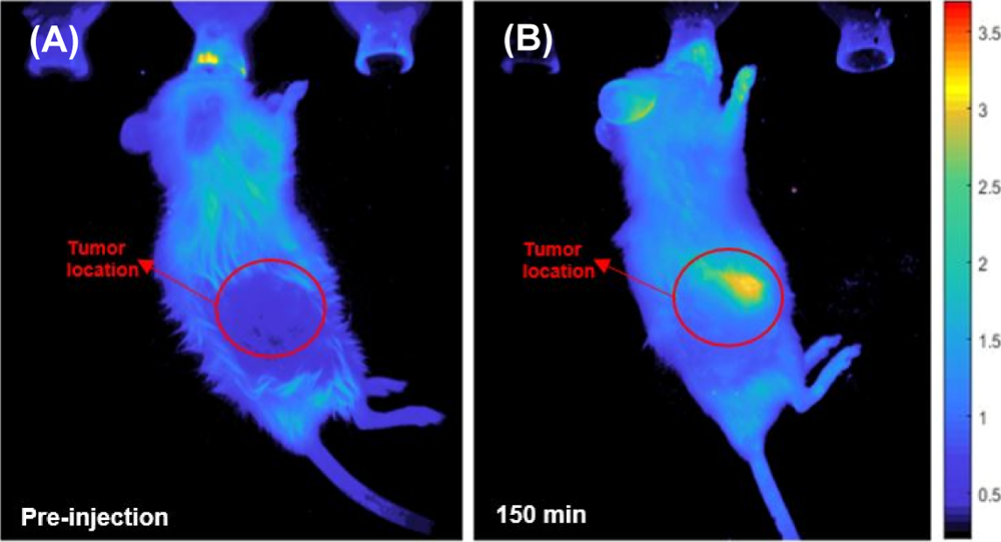
*In vivo* fluorescent images of mice bearing patient-derived WHIM4 tumor cells (up to 226.8 mm^3^ in volume) after intravenous injections of HA-nCQDs. The HA-nCQDs were detectable at the tumor site at 150 min postinjection.

In the second set of experiments, mice-bearing WHIM4 tumors were intravenously injected with either nCQDs or HA-nCQDs. As seen in Figure 10, 150 min after intravenous injection, carbon quantum dots had accumulated in the tumor when they were coated with hyaluronic acid. Noncoated carbon quantum dots, however, were not detected. This experiment further confirms the ability of HA-nCQDs to target CD44 receptors. Furthermore, the carbon quantum dots’ accumulation in the tumor was likely assisted by leaky vessels because permeability and tumor progress is closely linked.^59^

## Conclusions

In summary, we have synthesized HA-nCQDs and explored their potential in tumor imaging. The nCQDs and their conjugates fluoresce at different wavelengths depending upon their wavelength of excitation. Using HA-nCQDs, we observed enhanced internalization of carbon quantum dots, which was mediated by CD44 receptors. In an *in vivo* imaging study, we screened patient-derived tissue microarrays of major breast cancer subtypes and chose a relatively high-CD44 receptor-expressing model (WHIM4) to be injected into mice for *in vivo* imaging. The WHIM4-bearing mice were injected with either nCQDs or HA-nCQDs, either subcutaneously or intravenously. In both cases, coating carbon quantum dots with HA allowed them to accumulate more profoundly into the tumor. Our findings demonstrated that hyaluronic acid-coated carbon quantum dots could be used for *in vivo* tumor imaging and, potentially, as targeted drug delivery agents.

## Supporting Information Available

Particle size distribution, zeta potential analysis, and extinction coefficient measurement results of the carbon quantum dots, ARPE-19 and CHO cells’ viability, apoptotic cell amounts, and ROS generation responses in exposure to the carbon quantum dots, carbon quantum dots uptake assessment of ARPE-19 and CHO cells upon pretreatment with excess hyaluronic acid, laser scanning microscopy images of NIH 3T3 cells exposed to the carbon quantum dots, and comprehensive experimental details on the establishment and characterization of patient-derived tumor xenografts (PDF)
