# Supplementary material for: Hyaluronan-Conjugated Carbon Quantum Dots for Bioimaging Use
Source: ACS Appl Mater Interfaces. 2020 Dec 23;13(1):277–86. doi: 10.1021/acsami.0c20088 (PMC8243741; doi:10.1021/acsami.0c20088)
Supplement: Supplementary file 1 — am0c20088_si_001.pdf [file am0c20088_si_001.pdf]

Supporting Information

for

Hyaluronan-Conjugated Carbon Quantum Dots for Bioimaging Use

Bedia Begüm Karakoçak[1,2], Amine Laradji[1,2], Tina Primeau[3], Mikhail Y. Berezin[4], Shunqiang Li[3], and Nathan Ravi[1,2*]

[1]Department of Ophthalmology and Visual Sciences, Washington University in St. Louis, St. Louis, MO 63110, USA

[2]Veterans Affairs Medical Center, St. Louis, MO 63106, USA

[3]Department of Medicine, Washington University School of Medicine, St. Louis, MO 63110, USA

[4]Department of Radiology, Washington University School of Medicine, St. Louis, MO 63110, USA

Revised version

Submitted to

ACS Applied Materials & Interfaces

December 8, 2020

*Corresponding author
Tel.: +1 314-747-4458; Fax: +1 314-747-5073
E-mail: ravi@wustl.edu

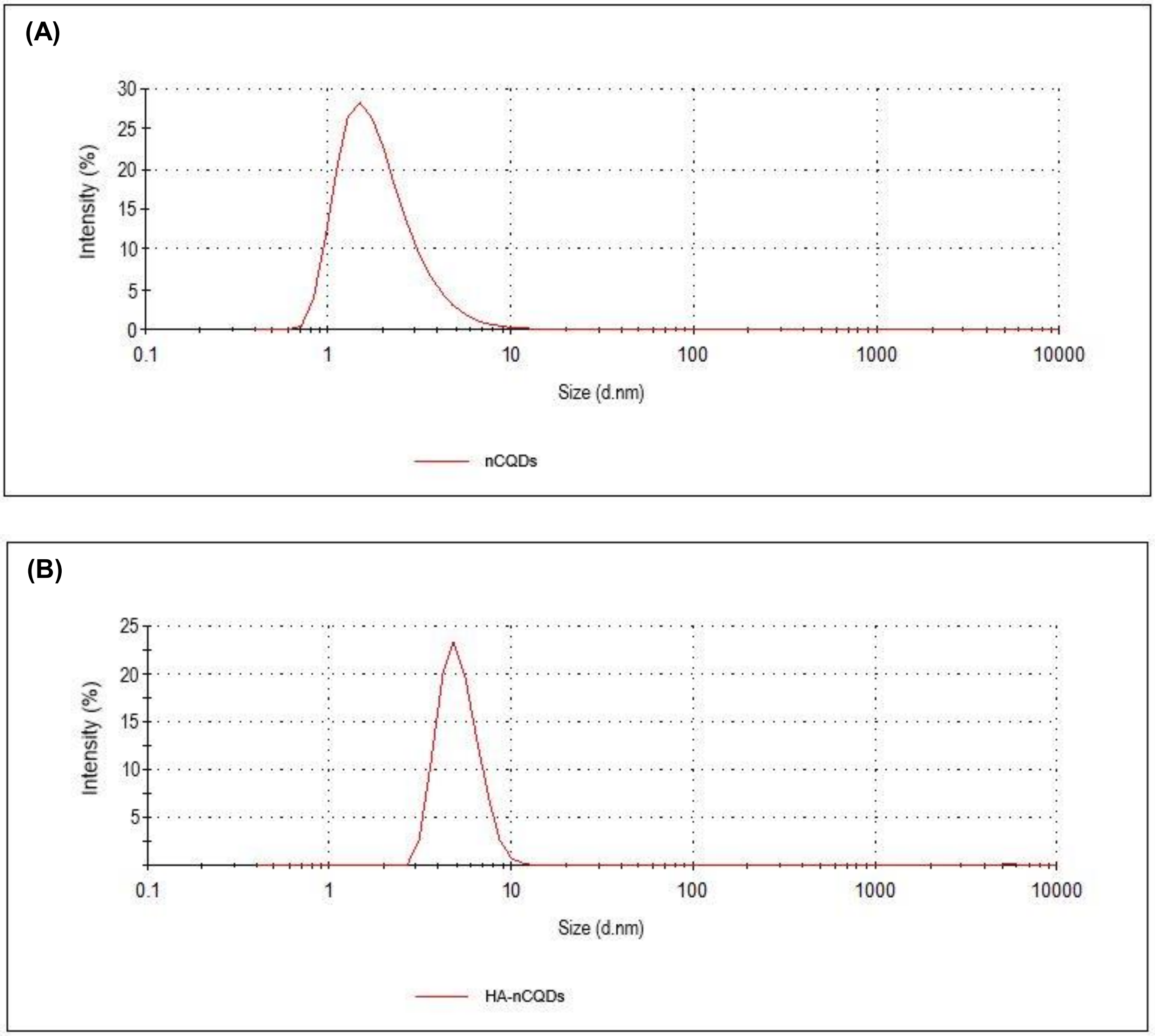

Figure S1. Raw size distributions (by intensity) of (A) uncoated carbon quantum dots (nCQDs) and (B) hyaluronic acid-conjugated carbon quantum dots (HA-nCQDs). The uncoated carbon quantum dots exhibited a hydrodynamic diameter of 2.01±1.95 nm with a PDI of 0.93, whereas HA-coated carbon quantum dots had a hydrodynamic diameter of 5.24±1.25 nm with a PDI of 0.06. The increase in size is attributed to the HA-coating.

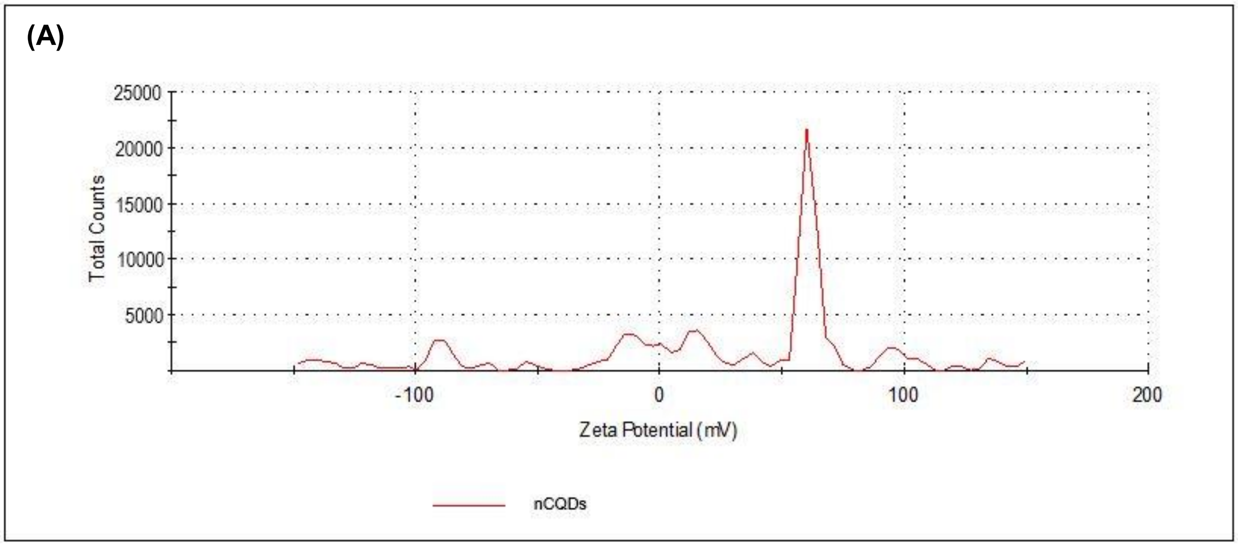

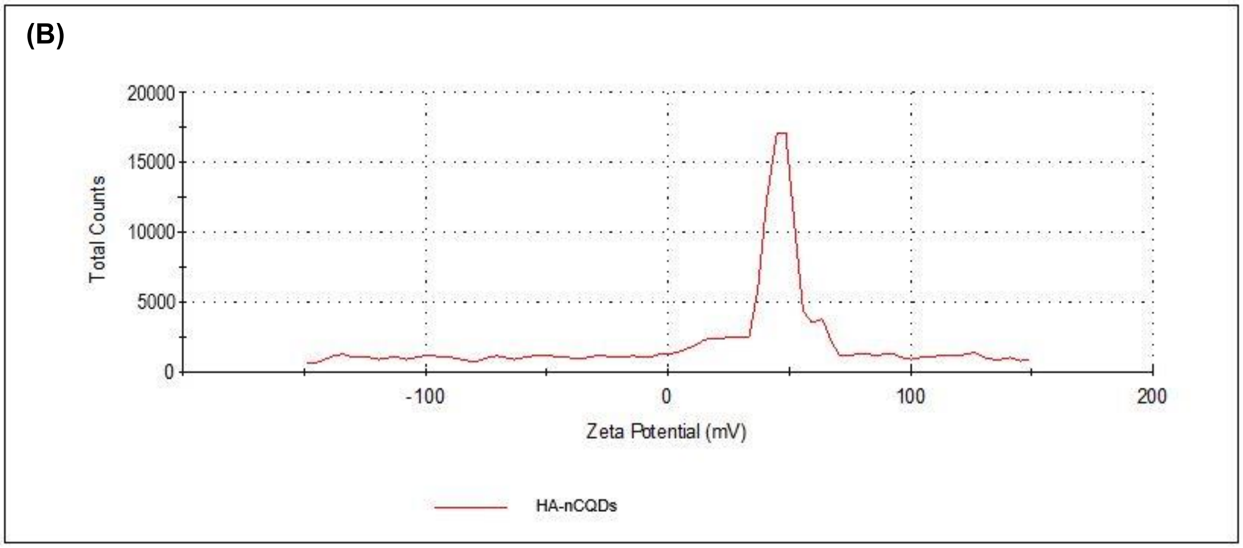

Figure S2. Raw zeta potential measurement results of (A) uncoated carbon quantum dots (nCQDs) and (B) hyaluronic acid-conjugated carbon quantum dots (HA-nCQDs). The uncoated carbon quantum dots exhibited a zeta potential of 61.3 mV, whereas HA-coated carbon quantum dots had a zeta potential of 44.8 mV. With the HA coating, although the zeta potential has decreased 27%, 44.8 mV is still considered a high enough potential for particles to repel each other. It is also noteworthy to add that visually no aggregates or precipitates were observed in the solution after the HA conjugation.

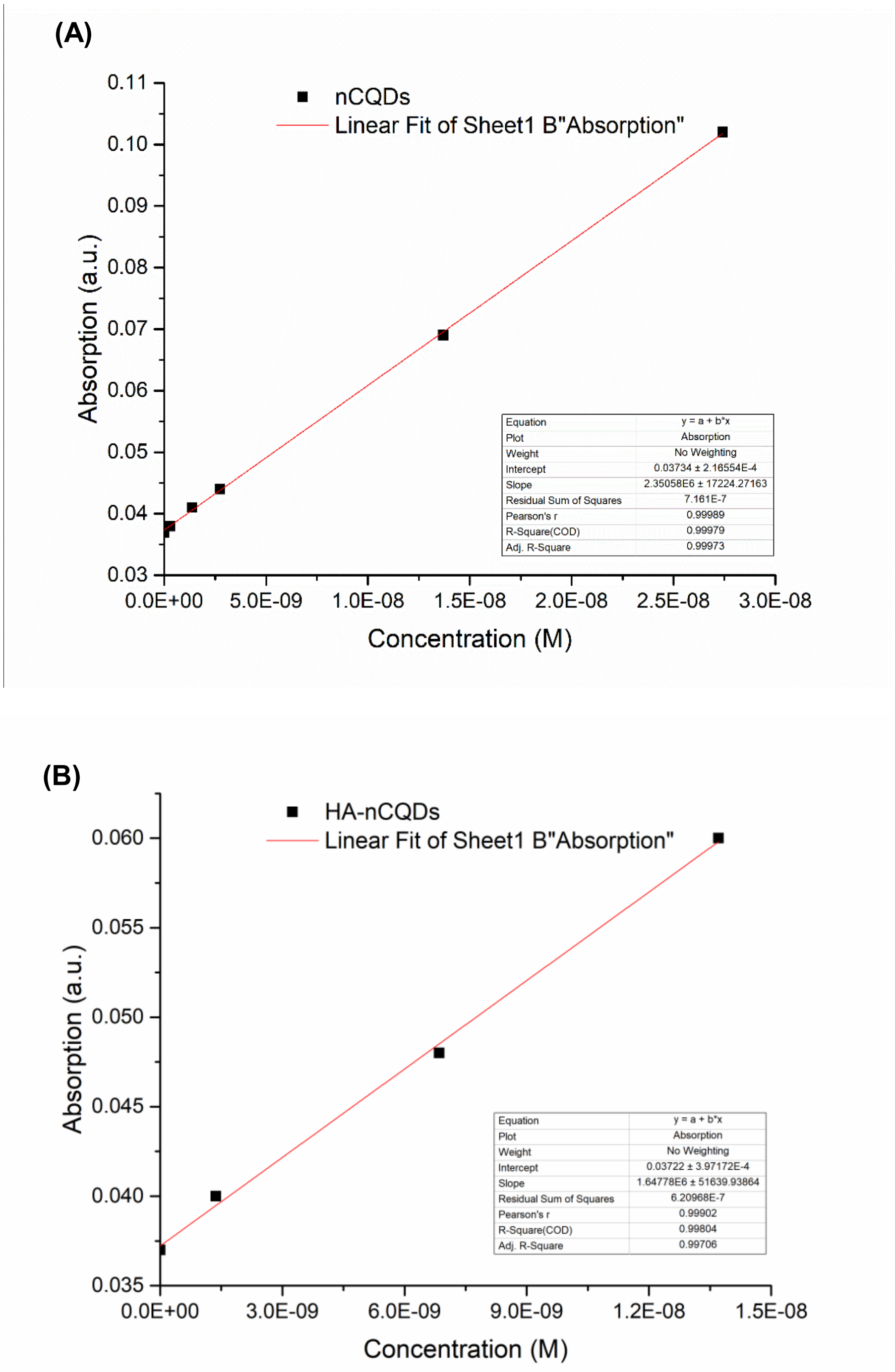

Figure S3. Extinction coefficient calculations results of (A) nCQDs and (B) HA-nCQDs in DI water. The slope of the curve corresponds to the extinction coefficient, ε ($M^{-1}cm^{-1}$). The ε values were 2.35×10^6 and 1.65×10^6 for nCQDs and HA-nCQDs, respectively. The results revealed that the extinction coefficient of HA-nCQDs was approximately 30% less than their unconjugated counterpart.

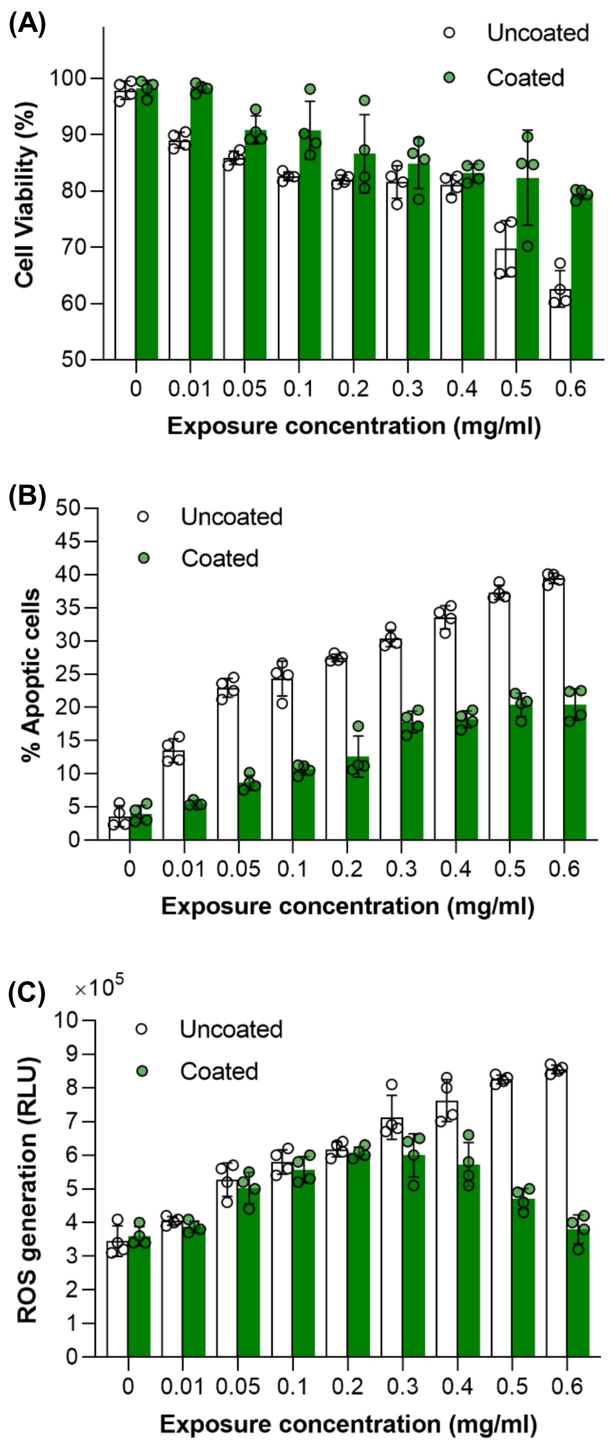

Figure S4. Biocompatibility results of nCQDs and HA-nCQDs with ARPE-19 cells. (A) % Viability (B) Apoptotic cell percentage (C) ROS generation measurement results after incubation with nCQDs for 24 h at concentrations up to 0.6 mg/mL. Data are expressed as mean ± SD (n=4, $^*P < 0.05$).

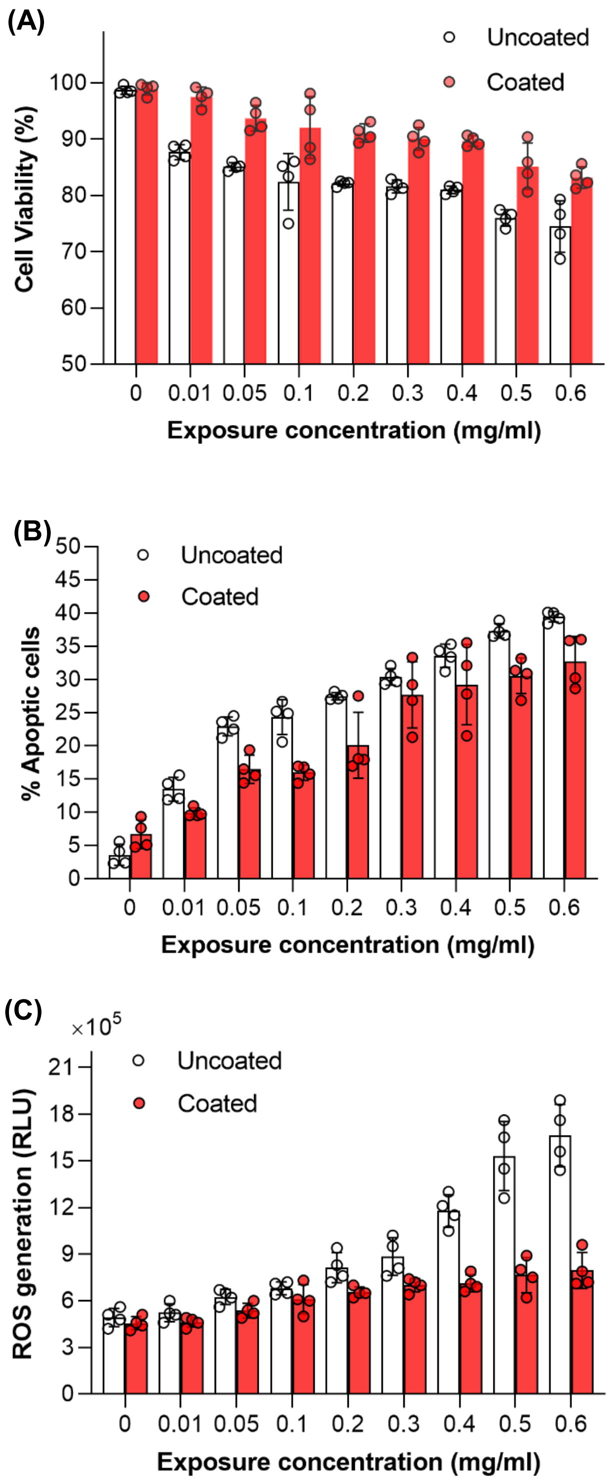

Figure S5. Biocompatibility results of nCQDs and HA-nCQDs with CHO cells. (A) % Viability (B) Apoptotic cell percentage (C) ROS generation measurement results after incubation with nCQDs for 24 h at concentrations up to 0.6 mg/mL. Data are expressed as mean ± SD (n=4, **P* < 0.05).

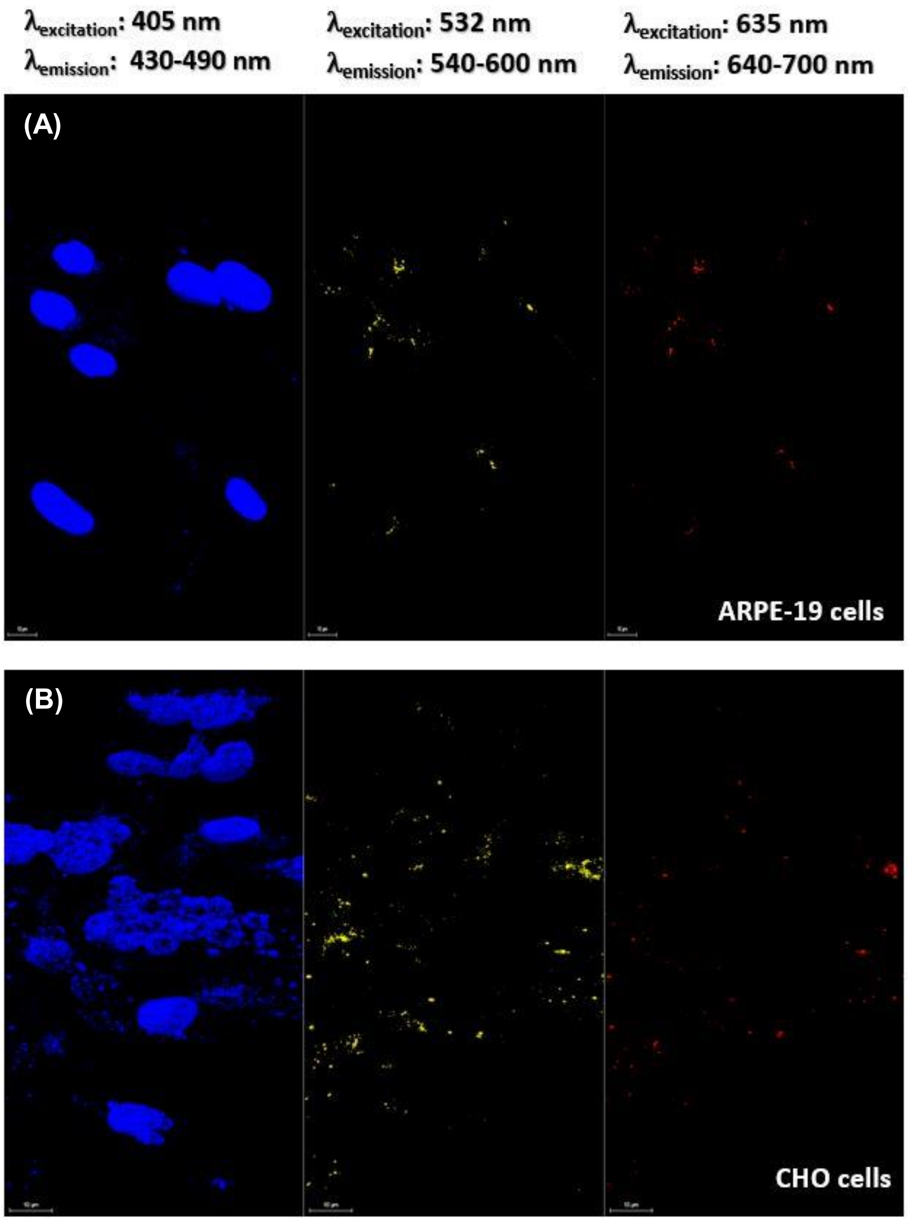

Figure S6. Confocal microscopy images of (A) Retinal pigment epithelial (B) CHO cells exposed to 0.6 mg/ml HA-nCQDs following the excess pretreatment of the cells with HA only. The excess HA pretreatment overloads the CD44 receptors; therefore, the internalized amount of HA-nCQDs decreases significantly. The cells were stained only for nuclei with DAPI. The scale bars are 10 μm.

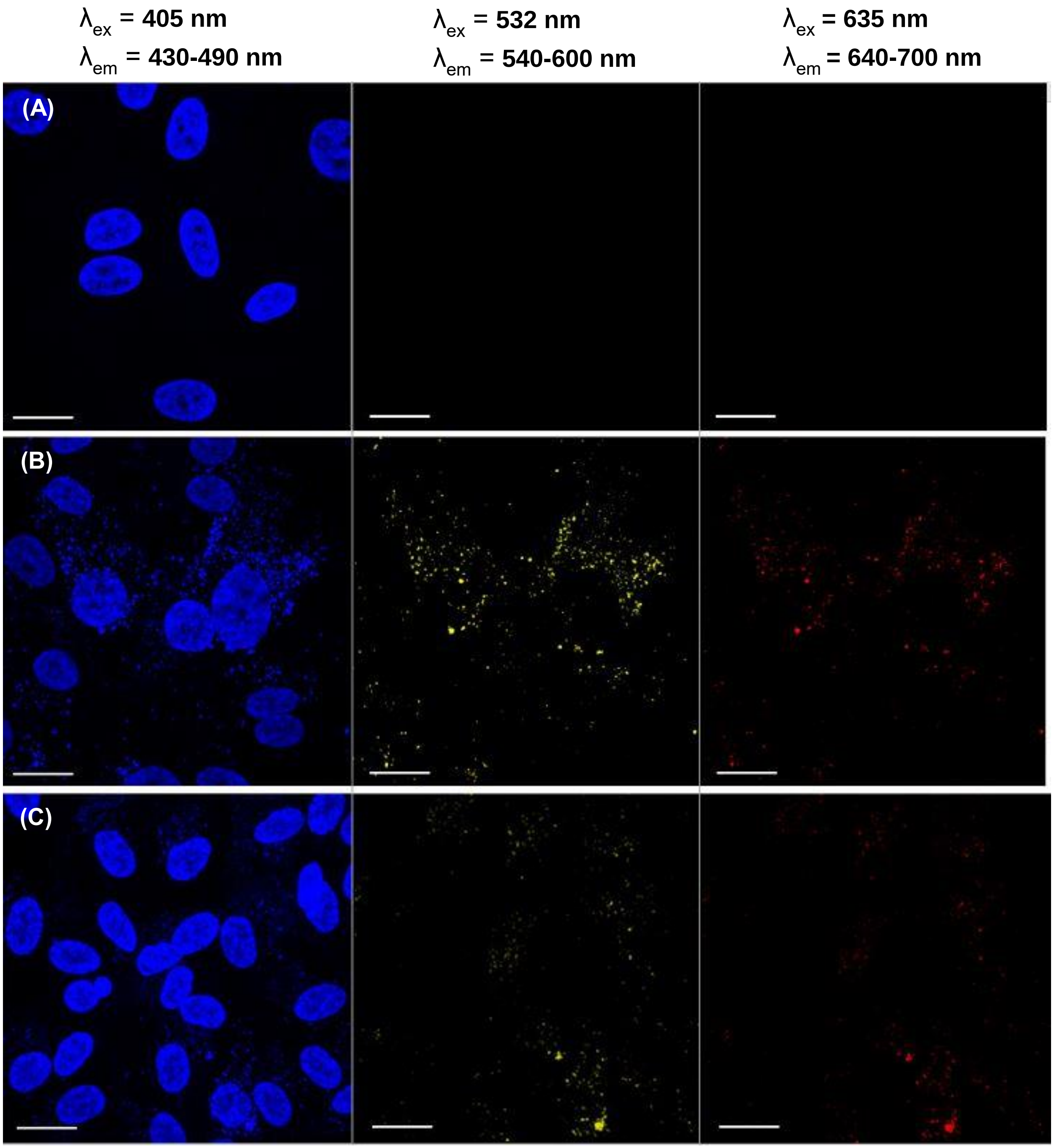

Figure S7. Confocal microscopy images of NIH 3T3 cells, which are known to have relatively less CD44 expression exposed to (A) no particles, control; (B) 0.6 mg/ml nCQDs; (C) 0.6 mg/ml HA-nCQDs. The cell nuclei were stained with DAPI. The scale bars are 20 µm. The number of nCQDs *vs.* HA-nCQDs internalized by the NIH 3T3 cells was not significantly enhanced, unlike the enhanced internalization trend with the HA-conjugation observed with ARPE-19 and CHO cells, which exhibit more CD44 expression. The scale bars are 20 µm.

Establishment and Characterization of Patient-Derived Tumor Xenografts

Cancer is a complex disease comprised of a spectrum of cancer subtypes with distinct clinical phenotypes, genetic anomalies, and therapeutic responsiveness. While cell-line derived cancer xenografts are frequently used for preclinical testing, the ability of these models to predict drug efficacy is limited due to the alterations caused by long-term *in vitro* culture, the lack of the source patient's clinical information, and their poor representation of the complexity present in cancer. At Washington University in St. Louis School of Medicine, in collaboration with Dr. Matthew Ellis and others, Dr. Shunqiang Li launched the HAMLET (**H**uman **a**nd **M**ouse **L**inked **E**valuation of **T**umors) project in 2006 to establish patient-derived xenograft (PDX) models using breast cancer tissue and to compare the similarities between the original tumors and their xenografts. The goals of this project are to (1) use WHIM (**W**ashington University **H**uman **i**n **M**ouse) tumor models to bridge the knowledge gap between breast cancer genome structure and function; (2) test the anti-cancer efficacy of new drugs; and (3) apply WHIM tumor models to the study of personalized cancer therapy.

Dr. Li has successfully established over 100 WHIM tumor models by engrafting patient breast cancer tissues into NOD/SCID mice. The WHIM tumor models have been characterized by (1) global gene expression, (2) array comparative genomic hybridization (aCGH), and (3) in some cases, whole-genome sequencing and reverse-phase protein array (RPPA). The WHIM models exhibit remarkable genetic and phenotypic similarities with the human tumor from which they were derived.[1, 2] They are "live" replicas of human tumors.

In this study, we screened 35 different WHIM tumor models (Table S1) and tonsil tissue as a control (Fig. S8) for CD44 expression with Confocal microscopy.

Table S1. The tissue microarray numbers and corresponding codes. The codes were created based on the model, passage number, and mouse number.

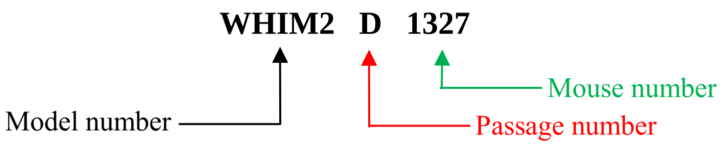

Tissue #	Tissue code
1	Tonsil Control
2	WHIM2D1327
**3**	**WHIM3E3222 (poor)**
**4**	**WHIM4C1347 (rich)**
5	WHIM5D3468
6	WHIM6G3290
7	WHIM10F2369
8	WHIM12C1375
9	WHIM13D1613
10	WHIM14D1574m
11	WHIM17E2939
12	WHIM21D1452m
13	WHIM22E2017
14	WHIM25E3485
15	WHIM29D2870
16	WHIM30F2602
17	WHIM31E2162
18	WHIM33E2105m
19	WHIM34G1969
20	WHIM36D3118
21	WHIM39E2871
22	WHIM41D3641m
23	WHIM42E3019
24	WHIM48D3114
25	WHIM53C3690m
26	WHIM55D3842
27	WHIM59E3484
28	WHIM61C3165
29	WHIM63E3457
30	WHIM65E3495
31	WHIM66E3326
32	WHIM67D3519
33	WHIM68E3838
34	WHIM69D3417
35	WHIM70E3538

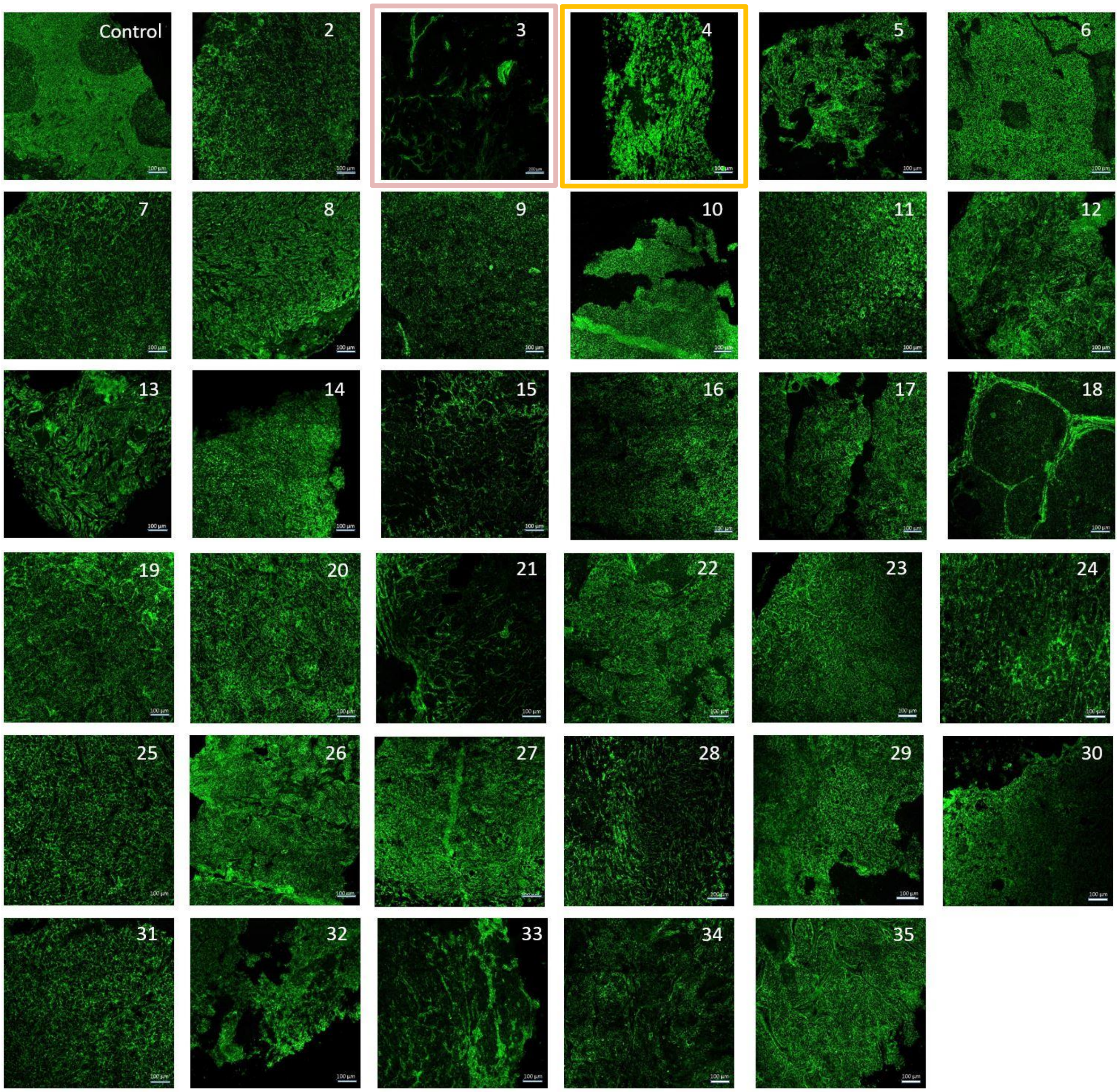

Figure S8. Confocal microscopy images of 34 different tissue microarray tissues and tonsil control. The cells were stained only for CD44 receptors with CD44 antibody. The poor-CD44-expressing and rich-CD44-expressing tissues are marked with pink and orange squares, respectively. The scale bars are 100 µm.

To further confirm the Confocal imaging analysis results, we chose one poor CD44-expressing and one rich CD44 expressing breast cancer cell type models (model 3 and model 4, respectively) and performed flow cytometry analysis (Fig. S9). The results showed that indeed, the model 4 (WHIM4) TMA express more CD44 receptors compared to model 3 (WHIM3).

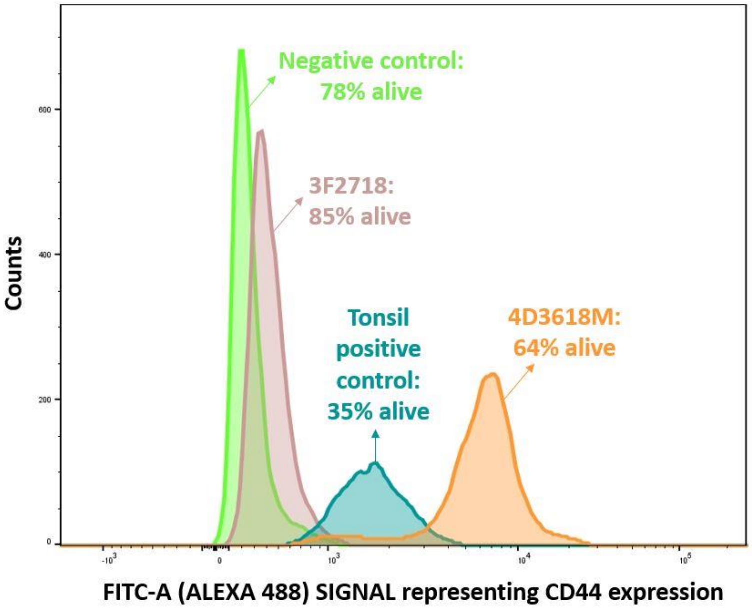

	Sample Name	Subset Name	Count
	[WHIM 4D3618M.fcs]	Alive	6407
	[TONSIL CONTROL.fcs]	Alive	3497
	[Specimen_001_WHIM 3F_002.fcs]	Alive	8474
	[Specimen_001_Negative Control Lens_001.fcs]	Alive	7804

Figure S9. Flow cytometry analysis of poor (model 3) and rich (model 4) CD44-expressing breast cancer cells and a tonsil control for CD44 expression.

References

1. Ding, L.; Ellis, M. J.; Li, S.; Larson, D. E.; Chen, K.; Wallis, J. W.; Harris, C. C.; McLellan, M. D.; Fulton, R. S.; Fulton, L. L.; Abbott, R. M.; Hoog, J.; Dooling, D. J.; Koboldt, D. C.; Schmidt, H.; Kalicki, J.; Zhang, Q.; Chen, L.; Lin, L.; Wendl, M. C.; McMichael, J. F.; Magrini, V. J.; Cook, L.; McGrath, S. D.; Vickery, T. L.; Appelbaum, E.; Deschryver, K.; Davies, S.; Guintoli, T.; Lin, L.; Crowder, R.; Tao, Y.; Snider, J. E.; Smith, S. M.; Dukes, A. F.; Sanderson, G. E.; Pohl, C. S.; Delehaunty, K. D.; Fronick, C. C.; Pape, K. A.; Reed, J. S.; Robinson, J. S.; Hodges, J. S.; Schierding, W.; Dees, N. D.; Shen, D.; Locke, D. P.; Wiechert, M. E.; Eldred, J. M.; Peck, J. B.; Oberkfell, B. J.; Lolofie, J. T.; Du, F.; Hawkins, A. E.; O'Laughlin, M. D.; Bernard, K. E.; Cunningham, M.; Elliott, G.; Mason, M. D.; Thompson, D. M., Jr.; Ivanovich, J. L.; Goodfellow, P. J.; Perou, C. M.; Weinstock, G. M.; Aft, R.; Watson, M.; Ley, T. J.; Wilson, R. K.; Mardis, E. R., Genome remodelling in a basal-like breast cancer metastasis and xenograft. *Nature* **2010,** *464* (7291), 999-1005.
2. Li, S.; Shen, D.; Shao, J.; Crowder, R.; Liu, W.; Prat, A.; He, X.; Liu, S.; Hoog, J.; Lu, C.; Ding, L.; Griffith, O. L.; Miller, C.; Larson, D.; Fulton, R. S.; Harrison, M.; Mooney, T.; McMichael, J. F.; Luo, J.; Tao, Y.; Goncalves, R.; Schlosberg, C.; Hiken, J. F.; Saied, L.; Sanchez, C.; Giuntoli, T.; Bumb, C.; Cooper, C.; Kitchens, R. T.; Lin, A.; Phommaly, C.; Davies, S. R.; Zhang, J.; Kavuri, M. S.; McEachern, D.; Dong, Y. Y.; Ma, C.; Pluard, T.; Naughton, M.; Bose, R.; Suresh, R.; McDowell, R.; Michel, L.; Aft, R.; Gillanders, W.; DeSchryver, K.; Wilson, R. K.; Wang, S.; Mills, G. B.; Gonzalez-Angulo, A.; Edwards, J. R.; Maher, C.; Perou, C. M.; Mardis, E. R.; Ellis, M. J., Endocrine-therapy-resistant ESR1 variants revealed by genomic characterization of breast-cancer-derived xenografts. *Cell Rep* **2013,** *4* (6), 1116-30.
